# Comparing the Efficacy of Carvedilol and Flecainide on the Treatment of Idiopathic Premature Ventricular Complexes from Ventricular Outflow Tract: A Multicenter, Randomized, Open-Label Pilot Study

**DOI:** 10.3390/jcm12082887

**Published:** 2023-04-15

**Authors:** Jongmin Hwang, Yong-Seog Oh, Hyoung-Seob Park, Jong-Il Choi, Young Soo Lee, Eue-Keun Choi, Dong-Gu Shin, Young Keun On, Tae-Hoon Kim, Hyung Wook Park, Min Soo Cho, Myung Hwan Bae, Seongwook Han

**Affiliations:** 1 Cardiovascular Center, Keimyung University Dongsan Hospital, Daegu 42601, Republic of Korea; dsmcep@gmail.com (J.H.);; 2 Division of Cardiology, Department of Internal Medicine, Seoul St. Mary’s Hospital, The Catholic University of Korea, Seoul 06591, Republic of Korea; 3 Arrhythmia Center, Korea University Medical Center Anam Hospital, Seoul 02841, Republic of Korea; 4 Department of Cardiology, Catholic University of Daegu, Daegu 42472, Republic of Korea; 5 Department of Internal Medicine, Seoul National University Hospital, Seoul 03080, Republic of Korea; 6 Division of Cardiology, Department of Internal Medicine, Yeungnam University Medical Center, Yeungnam University College of Medicine, Daegu 42415, Republic of Korea; 7 Division of Cardiology, Department of Internal Medicine, Heart Vascular Stroke Institute, Samsung Medical Center, Sungkyunkwan University School of Medicine, Seoul 06351, Republic of Korea; 8 Division of Cardiology, Department of Internal Medicine, Severance Cardiovascular Hospital, Yonsei University College of Medicine, Seoul 03722, Republic of Korea; 9 Department of Cardiology, Chonnam National University Hospital, Gwangju 61469, Republic of Korea; 10 Department of Cardiology, University of Ulsan College of Medicine, Asan Medical Center, Seoul 05505, Republic of Korea; 11 Division of Cardiology, Department of Internal Medicine, Kyungpook National University Hospital, Daegu 41944, Republic of Korea

**Keywords:** ventricular premature complexes, carvedilol, carvedilol, anti-arrhythmia agents

## Abstract

The mechanism of premature ventricular complexes (PVC) occurring in the ventricular outflow tract (OT) is related to an intracellular calcium overload and delayed afterdepolarizations that lead to triggered activity. The guidelines recommend using beta-blockers and flecainide for idiopathic PVCs, but they also acknowledge the limited evidence supporting this recommendation. We conducted a multicenter, randomized, open-label pilot study comparing the effect of carvedilol and flecainide on OT PVC, which are widely used to treat this arrhythmia. Patients with a 24 h Holter recording a PVC burden ≥ 5%, which showed positive R waves in leads II, III, and aVF, and without structural heart disease were enrolled. They were randomly assigned to the carvedilol or flecainide group, and the maximum tolerated dose was administered for 12 weeks. A total of 103 participants completed the protocol: 51 with carvedilol and 52 with flecainide. After 12 weeks of treatment, the mean PVC burden significantly decreased in both groups: 20.3 ± 11.5 to 14.6 ± 10.8% with carvedilol (*p* < 0.0001) and 17.1 ± 9.9 to 6.6 ± 9.9% with flecainide (*p* < 0.0001). Both carvedilol and flecainide effectively suppressed OT PVCs in patients without structural heart disease, with flecainide showing a superior efficacy compared to carvedilol.

## 1. Introduction

Premature ventricular complexes (PVC) occurring in a structurally normal heart are called idiopathic PVCs, and they commonly originate from the ventricular outflow tract (OT) myocardium [1,2]. OT PVCs often need treatment due to their disruptive symptoms and PVC-induced cardiomyopathy [3]. Although catheter ablation technology is developing remarkably, pharmacological treatment is still the cornerstone treatment of OT PVCs. Beta-blockers or non-dihydropyridine calcium channel blockers are recommended as the first-line treatment by recent guidelines and help reduce recurrent arrhythmias and improve symptoms [2,3]. However, most studies that examined the antiarrhythmic effects of beta-blockers on idiopathic PVCs are outdated and mainly used first- and second-generation beta-blockers [4].

Carvedilol is one of the most effective beta-blockers for reducing ventricular arrhythmias and mortality in patients with heart failure [5,6]. In addition to its nonselective beta-blockade effects, its antioxidative and alpha-blockade effects have been proposed as the mechanisms behind the antiarrhythmic property of carvedilol. Recently, the inhibition of a store overload-induced calcium release (SOICR) has been suggested to be one of the antiarrhythmic mechanisms of carvedilol [7]. The SOICR causes intracellular calcium overload, activating the Na^+^/Ca^2+^ exchangers and finally resulting in delayed afterdepolarizations (DADs) that lead to triggered activity. Among the various beta-blockers, only carvedilol is known to have the potential to directly inhibit the release of SOICR in vitro [7,8]. The mechanism of OT PVC formation is considered a triggered activity [9], and triggered activity is closely related to a sarcoplasmic reticulum (SR) calcium overload, DAD, and SOICR.

We hypothesized that carvedilol would be more effective for OT PVCs than other beta-blockers due to its direct SOICR inhibition effect. Therefore, we aimed to demonstrate its effect through a multicenter, randomized, open-label pilot study. In addition, a randomized, direct comparison with flecainide was planned. Although flecainide is a well-proven class Ic antiarrhythmic agent and is already widely used for treating OT PVCs, there has been no structured, randomized trial to demonstrate its efficacy for OT PVCs.

## 2. Materials and Methods

### 2.1. Study Design

This was a multicenter, randomized, open-label pilot study comparing the effect of carvedilol with flecainide on OT PVCs conducted in 11 tertiary hospitals in South Korea. Data collection adhered to the principles outlined in the Declaration of Helsinki (2013) and Good Clinical Practices. The ethics committees and corresponding health authorities approved the protocol for all sites. All the patients provided written informed consent before enrollment. This trial was registered with ClinicalTrials.gov, number NCT03587558.

### 2.2. Patient Selection and Randomization

Individuals who met the following criteria were included in the study: (1) between the ages of 19 and 80 years with no previous history of treatment for PVCs; (2) had symptomatic OT PVC, which was confirmed by positive R waves in leads II, III, and aVF of electrocardiography (ECG); (3) over 5% of PVC burden (the amount of PVCs reported as the % of beats of ventricular origin of the total amount of beats over a 24 h Holter recording period) [10]; (4) normal left ventricular (LV) ejection fraction (EF) (≥50%) without known structural heart disease; and (5) to exclude catecholaminergic polymorphic ventricular tachycardia (CPVT), a treadmill test was performed in all patients before enrollment. Exclusion criteria included having any structural heart disease with or without obstructive coronary arterial disease, a history of asthma or chronic obstructive pulmonary disease, systolic blood pressure below 90 mmHg, and any form of significant bradyarrhythmia. Atrial fibrillation patients were also excluded. Detailed exclusion criteria are shown below:

(1) Pregnant subjects, subjects planning for pregnancy, or breastfeeding women (nursing mothers);
(2) History of coronary artery disease;
(3) History of hypersensitivity to carvedilol;
(4) Subjects with cardiogenic shock;
(5) Subjects with a significant atrioventricular block (AV block) (2nd or 3rd degree) or sinoatrial node dysfunction;
(6) Subjects with cardiomyopathy with or without reduced LV EF;
(7) Presence or history of respiratory diseases with bronchospasms, such as asthma and chronic obstructive pulmonary disease;
(8) Receiving any medications known as monoamine oxidase inhibitors;
(9) Subjects with severe hypotension displaying systolic blood pressure ≤ 90 mmHg;
(10) Subjects with severe hepatic impairment;
(11) Asymptomatic PVC or non-sustained ventricular tachycardia (NSVT) following myocardial infarction;
(12) Subjects with any atrial fibrillation.

Randomization was performed by an independent statistician at a ratio of 1:1 based on the sequence and was stratified according to the trial site using the SAS system randomization program (SAS software, version 9.2; SAS Institute Inc., Cary, NC, USA).

### 2.3. Treatment and Follow-Up

Eligible patients were randomized into either the carvedilol group or the flecainide group. Participants assigned to the carvedilol group received a once-daily dose of a sustained-release formulation of carvedilol. In South Korea, an 8/16/32/64 mg sustained-release formulation, once-daily dose of carvedilol is available and equivalent to 3.125/6.25/12.5/25 mg twice daily. Participants assigned to the flecainide group received a twice-daily dose of flecainide as the conventional method. The dosages of the medications were titrated up to the maximum based on the patient’s tolerance and the occurrence of side effects, which were evaluated every two weeks. The assessment of whether the dosage of the drug has reached the maximum was left to each physician’s discretion.

The patient’s tolerance and occurrence of hypotension and bradycardia were mainly assessed in the carvedilol group. In the flecainide group, QRS widening was intensively monitored. After reaching the maximum tolerated dose, the drug was maintained for three months. Baseline clinical and demographic data were collected along with the patients’ 24 h Holter monitoring data. Interim outpatient clinic visits were also left to the physician’s discretion, but the following schedule was recommended:

(1) Visit 1 (Baseline): Informed consent was achieved, and confirmation of inclusion/exclusion criteria with randomization was performed. Information including body weight, gender, age, name (initials), and concomitant diseases was collected from subjects. Physical examination and heart rate/blood pressure measurement while sitting were carried out. The results of routine 12-lead ECG, echocardiography, treadmill test, and PVC burden on 24 h Holter monitoring were investigated. Patient-reported symptoms, assessed on a visual analog scale (ranging from 0 to 10, with higher scores indicating a greater intensity of discomfort, VAS), were also recorded.
(2) Visit 2 (2 weeks ± 7 days): The following data were collected: heart rate and blood pressure while sitting, routine 12-lead ECG, VAS score, and medication compliance. Patients were asked whether any adverse events had occurred and to grade the event, if any.
(3) Visit 3 (4 weeks ± 7 days) and Visit 4 (6 weeks ± 7 days): These visits were optional. However, according to the attending doctor’s discretion, more frequent visits could be made for drug dosage optimization.
(4) Visit 5 (3 months ± 7 days from when a subject reaches their maximum tolerable dose): The measurement of the heart rate/blood pressure while sitting was carried out. In addition, the results of routine 12-lead ECG and PVC burden on 24 h Holter monitoring were collected. Patient-reported symptoms, assessed on a VAS, were also recorded.

Any of the following medications were not taken during the study period:

(1) Antihypertensives other than carvedilol, especially the following drugs:
  ① Beta-blockers;
  ② Non-dihydropyridine calcium channel blockers: diltiazem, verapamil;
(2) Monoamine oxidase inhibitors;
(3) Antiarrhythmic drugs, including digitalis;
(4) Other drugs that might affect patient safety or analysis of the study’s outcome in the opinion of the investigator.

### 2.4. Outcomes

The primary outcome was an absolute reduction in the PVC burden after three months of treatment. The secondary outcome was patient-reported symptoms assessed on a VAS. The primary and secondary outcomes were compared between the carvedilol and flecainide groups. The safety outcomes were assessed at each visit and included participant-reported adverse events. A serious adverse event was defined as any medical event that resulted in death, was life-threatening, required hospitalization, or caused substantial disability or incapacity. A severe adverse event was any event that interfered with the patient’s usual function, as deemed by the investigator on the case report form.

### 2.5. Statistical Analysis

Until now, no randomized study has evaluated the efficacy of carvedilol and flecainide on OT PVCs. Therefore, we conducted a pilot study, and sample size calculation was not performed. The researchers aimed for 100 participants (about 50 patients for each group) because it was felt that this would be a large enough sample for the pilot trial.

Continuous variables are expressed as the mean value ± standard deviation or interquartile range when the values do not follow a normal distribution. Categorical variables are expressed as numbers and percentages. The paired/independent sample *t*-test and chi-square test were used for continuous and categorical variables if normality was accepted. If the sample did not meet the normality assumption, the following method was used: the Mann–Whitney test was used to compare within-group continuous variables before and after the intervention, and the Wilcoxon signed-rank test was used to analyze the differences and changes in values between the two groups. Statistical analyses were performed using the MedCalc Statistical Software (version 20.015 MedCalc Software Ltd., Ostend, Belgium) or SAS (version 9.2 or newer; SAS Institute). A *p*-value < 0.05 was considered statistically significant.

### 2.6. Role of the Funding Source

The corresponding author designed the trial. The sponsor, Chong Kun Dang Pharmaceutical Corporation, did not contribute to the trial design, conduct, oversight, data analysis, or manuscript writing. The first two authors and the corresponding author mainly wrote the manuscript. All authors reviewed and approved the final draft. The sponsor covered all costs associated with the trial, including the cost of the carvedilol and all tests that were not otherwise clinically indicated for the trial’s purposes. Most data analyses were performed by a clinical research organization (Seoul CRO). All events were documented from sources, including, but not limited to, the paper and electronic charts, the laboratory, and imaging test reports, and were adjudicated by an independent clinical events committee, whose members were unaware of the trial-group assignments. Serious adverse events were reviewed by an independent data and safety monitoring board according to a predefined schedule.

## 3. Results

### 3.1. Study Population

From 20 June 2018 through 9 June 2020, 103 patients were recruited and randomly assigned to the carvedilol (*n* = 51) or the flecainide (*n* = 52) groups. The participants’ baseline demographic and clinical characteristics are shown in Table 1, and there were no statistical differences between the two groups. No patients were suspected of having CPVT in the treadmill test.

Most patients in the carvedilol group received an 8 mg (41.2%) or 16 mg (52.9%) dose, and in the flecainide group, 69.2% received 100 mg and the rest received 150 mg or 200 mg (28.8%). Blood pressure, heart rate, PR interval, and QRS duration changes after treatment are shown in Table 2. The carvedilol group showed no significant change in blood pressure before and after drug treatment. However, there was a substantial decrease in heart rate and a significant increase in PR interval. In the flecainide group, there was no change in blood pressure before and after treatment and no change in heart rate. However, there was a significant increase in PR interval and QRS duration.

### 3.2. Primary Outcome

**Figure 1:**
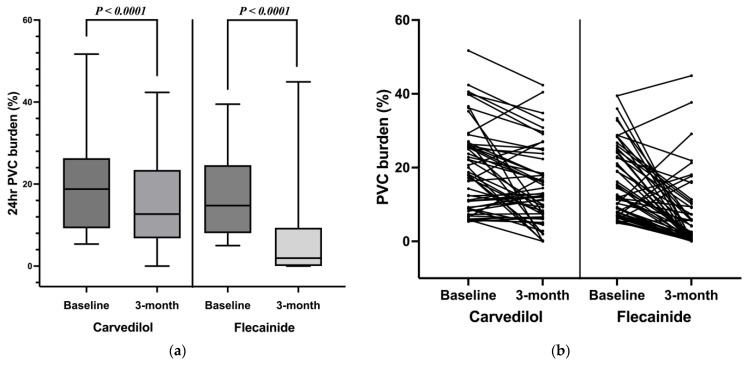
Effect of carvedilol and flecainide on premature ventricular complex burden before and after three months of treatment: (**a**) box plots, (**b**) individual responses shown in spaghetti plots.

**Figure 2:**
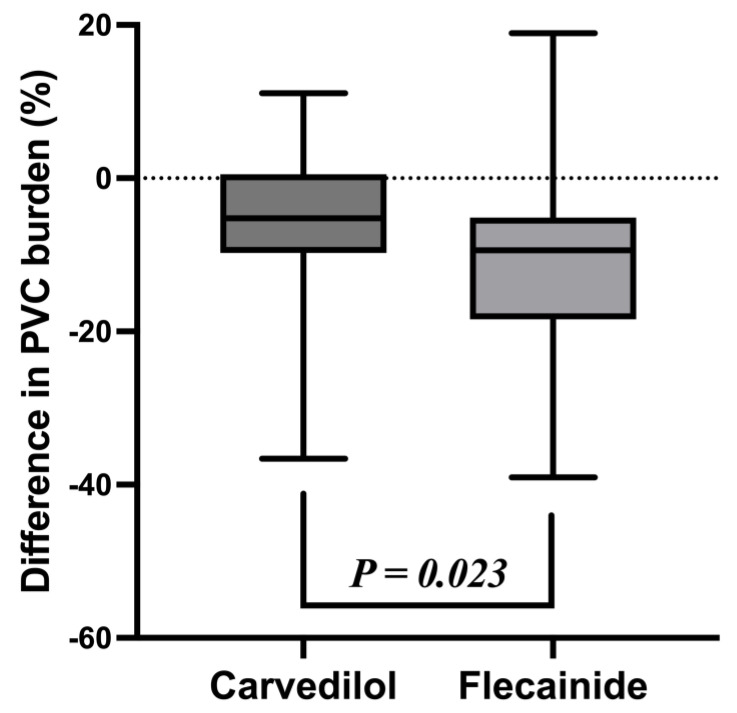
The mean difference of each group in the premature ventricular complex burden before and after three months of treatment.

After three months of treatment with the maximum tolerated dose, the PVC burden on the 24 h Holter monitor decreased significantly in both groups: 20.3 ± 11.5 to 14.6 ± 10.8% in the carvedilol group (*p* < 0.0001) and 17.1 ± 9.9 to 6.6 ± 9.9% in the flecainide group (*p* < 0.0001) (Figure 1). The mean difference in PVC burden before and after the treatment in each group was carvedilol −5.6 ± 9.3% and flecainide −10.6 ± 12.1%. Flecainide was more effective than carvedilol in reducing the PVC burden: the difference between the groups before and after the treatment was −4.9% (95% confidence interval [CI], −9.15 to −0.68, *p* = 0.023) (Figure 2).

The used dosages and the efficacy on PVC reduction according to each dosage of the drugs are shown in Table 3.

### 3.3. Secondary and Safety Outcome

**Figure 3:**
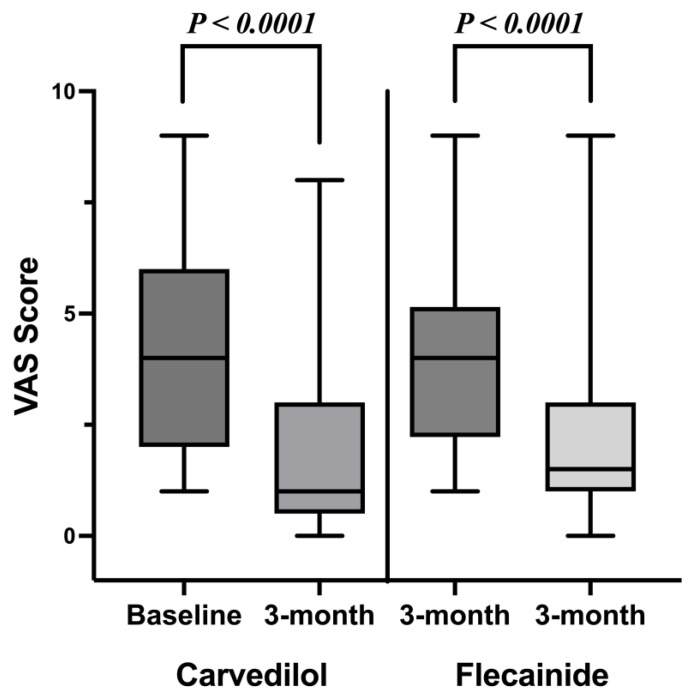
Effect of carvedilol and flecainide on symptoms of premature ventricular complexes before and after three months of treatment.

The secondary outcome, symptom improvement, was assessed by a 10-item VAS scale, and it also decreased significantly in both groups after the treatment: 4.2 ± 2.1 to 2.1 ± 2.2% in the carvedilol group (*p* < 0.0001) and 4.1 ± 2.2 to 2.0 ± 2.0% in the flecainide group (*p* < 0.0001) (Figure 3). The degree of symptom improvement did not differ between the two groups (*p* = 0.685). The major results of the study are summarized in Table 4.

No serious adverse events were observed in either treatment group during the study period. Minor adverse events in the carvedilol group were as follows (*n* = 51): two involving dizziness, two involving dyspnea on exertion, one involving a headache, one involving fatigue, one involving palpitation, and one involving sexual dysfunction. In the flecainide group, minor adverse events were as follows (*n* = 52): one involving dizziness, two involving headaches, one involving a burning sensation, one involving somnolence, one involving fatigue, and one involving blurred vision.

## 4. Discussion

### 4.1. Main Findings

In our study, carvedilol effectively reduced the PVC burden and symptoms of OT PVCs: the PVC burden decreased by an average of 5.6%, and the VAS symptom score was reduced by half after three months of carvedilol treatment. Flecainide, selected as a control, also effectively suppressed OT PVCs: the PVC burden was decreased by an average of 10.6%, and the VAS symptom score was also reduced by half. During the three months of treatment, no clinically significant side effects were observed in both groups.

### 4.2. Mechanism of OT PVCs

Lerman is one of the pioneers in this field, having elegantly demonstrated that cAMP-mediated DAD and triggered activity are the crucial mechanisms of OT PVCs [9,11]. Clinically, OT PVCs often appear or worsen due to physical and mental stress, suggesting that adrenergically mediated mechanisms play a significant role in their development. In the electrophysiology laboratory, OT PVCs can be elicited by administering isoproterenol/atropine infusion, burst pacing, or a combination of these approaches. In addition, they can be terminated with overdrive pacing or the administration of intravenous beta-blockers, calcium-channel blockers, or adenosine. All of these findings are evidence that OT PVCs are closely related to sympathetically/adrenergically mediated activation.

The sympathetic response is initiated by a release of norepinephrine from the cardiac sympathetic nerves and epinephrine from the adrenal medulla, which binds to β-adrenergic receptors on cardiomyocytes. This triggers a signaling cascade, leading to an increase in cAMP and consequent protein kinase A activation and phosphorylation of a myriad of targets, including the L-type calcium channel, type 2 ryanodine receptor (RyR2), and phospholamban [12]. These responses increase intracellular calcium concentrations, which induce positive chronotropy, inotropy, lusitropy, and dromotropy.

However, in case of excess intracellular calcium concentration, there is increased activity of the SR calcium pumps (SERCA). The SERCA increases its activity to remove calcium from the cardiac cellular myoplasm into the SR. When the SR reaches a critical threshold, it cannot accommodate such calcium overload and spontaneously releases some of the calcium back into the myoplasm through RyR2 after repolarization and during diastole; this process is called SOICR [13]. The elevated diastolic calcium in the myoplasm induces an electrogenic sodium–calcium exchanger to transport three sodium ions into the cell for every calcium ion extruded. The transient inward sodium current results in a DAD during phase 4 of the action potential. If the DAD is of sufficient amplitude, a new action potential is triggered, which can, in turn, generate successive action potentials called triggered activity. Therefore, increased calcium concentration in myoplasm and SOICR is the key mechanism of DAD and OT PVCs.

These hypothesized mechanisms are supported by the fact that when using therapeutic agents that antagonize the mechanisms, OT PVCs are suppressed. Beta-blockers terminate tachycardia by antagonizing the arrhythmogenic effects of adrenergic stimulation. Calcium-channel blockers diminish the slow inward calcium current and thus terminate the arrhythmia. Valsalva maneuvers release acetylcholine, which inhibits the production of adenylyl cyclase and cAMP, thereby lowering the level of intracellular calcium. Adenosine acts in a similar manner to acetylcholine to reverse intracellular calcium overload [9]. Given that adenosine has no antiarrhythmic effect in re-entry VT and only transiently suppresses (but does not terminate) automatic VT, its termination of outflow tract VT is considered pathognomonic for clinically identifying cAMP-mediated triggered activity [11].

### 4.3. Study Results Regarding Treatment of Symptomatic OT PVCs

PVCs are extremely common, and we frequently encounter patients with symptomatic OT PVCs in the clinic. As with previous guidelines, the latest guidelines also recommended the use of beta-blockers and flecainide for the treatment of idiopathic PVCs. However, guidelines also indicated that the evidence of this medical treatment is scarce [14]. Most studies were out-of-date and used first- and second-generation beta-blockers. In particular, there were no randomized controlled trials on treating OT PVCs with the third-generation beta-blockers that are widely used these days. To our knowledge, our study is the first prospective, randomized controlled trial evaluating the effects of carvedilol and flecainide in patients with symptomatic idiopathic OT PVCs.

Recently, Tang and colleagues published a paper regarding medical therapy for frequent idiopathic PVCs [15]. This study was similar to our research in that idiopathic PVCs were the primary study subject, but it was a prospective observational study, and bisoprolol was mainly used. In addition, data on patients using antiarrhythmic drugs were also analyzed, in which approximately 40% of patients were administered Class III antiarrhythmic drugs: 17 patients (63.0%) were on flecainide and 10 patients were on Class III drugs (6 on sotalol and 4 on amiodarone). Patients with PVCs of other origins were also enrolled. Therefore, their research and our research results should be evaluated independently. Nonetheless, the results were similar. They have shown that beta-blockers/calcium channel blockers reduced 30.5% of the PVC burden and Class I/III antiarrhythmic drugs reduced PVC burden by 81.3%. In our study, carvedilol reduced and flecainide reduced 28% and 62% of the PVC burden, respectively.

On the other hand, a retrospective study comparing RFA to medical therapy over a follow-up period of 6–12 months demonstrated reductions in PVC burden of 36% with beta-blockers and of 82% with antiarrhythmic drugs [16]. This study also focused on treating idiopathic PVC, similar to our research. However, the study included various sites of origin of idiopathic PVC, the types of beta-blockers were not specified, and there were only 9 patients in the flecainide group and 22 patients in the propafenone group. However, the results of this study were also similar to our results in that beta-blockers reduced 36% of the PVC burden and flecainide showed a reduction of 83%.

### 4.4. Antiarrhythmic Effect of Carvedilol

Carvedilol seems to have a more significant antiarrhythmic potential than the other beta-blockers [5,6,17]. Kanoupakis et al. showed that a 2-month carvedilol treatment in stable congestive heart failure patients significantly prolonged the atrial and ventricular effective refractory periods compared to a placebo [18]. The antioxidant and alpha-blocking activities of carvedilol have been suggested to contribute to its beneficial effects, but those were not corroborated by clinical studies [19,20]. Recently, the inhibition of SOICR through RyR2 has been suggested as an antiarrhythmic effect of carvedilol [7]. As described above, increased cytosolic Ca^2+^ can activate the inward Na^+^-Ca^2+^ exchange current, causing a DAD [21]. Indeed, SOICR-evoked DADs cause CPVT, which is associated with naturally occurring RyR2 mutations [22,23]. Among the various beta-blockers, only carvedilol is known to be a drug that can directly inhibit SOICR along with the beta-blockade effect [7]. Hence, carvedilol was introduced by a recently published position paper as a new approach to antiarrhythmic drug development that can prevent abnormal calcium handling as a direct RyR2 blocker [24]. With this knowledge, we hypothesized that carvedilol, which can reduce DAD by inhibiting RyR2 and SOICR, would be effective for OT PVCs.

Carvedilol is a non-selective beta-blocker, and its SOICR inhibitory effect has been experimentally proven. It has shown a superior antiarrhythmic effect compared to metoprolol in large-scale clinical studies of heart failure. Therefore, we assumed that carvedilol would show a distinguished efficacy on OT PVC inhibition compared to other beta blockers. However, the results of our study using carvedilol were not much different from the results of the observational study using bisoprolol [15] or the Mayo Clinic’s beta-blocker retrospective study published in 2014 [25].

### 4.5. Carvedilol and Flecainide for Treatment of OT PVCs

In our study, after three months of treatment, PVC burden was reduced by about 60% with flecainide and 25% with carvedilol. In particular, flecainide significantly suppresses OT PVCs from a dosage of 50 mg bid, and at higher dosages, it almost completely inhibits OT PVCs. Hence, flecainide showed superior antiarrhythmic effects, which was expected since it is a well-proven class Ic antiarrhythmic agent. However, one of our study goals was to verify the antiarrhythmic effect of carvedilol as described above, and contrary to our expectations, carvedilol showed only modest effects.

Although carvedilol did not show the efficacy that we expected in treating OT PVCs, it is certain that it significantly reduced the burden and symptoms of OT PVCs. The clinical meaning of our study can be summarized as follows. Firstly, no studies have systematically evaluated the treatment of idiopathic OT PVCs using carvedilol and flecainide. Furthermore, no studies have compared these two drugs in a randomized fashion. Through our study, the effects of carvedilol and flecainide on idiopathic OT PVCs were quantitatively and qualitatively evaluated. Second, carvedilol has well-proven clinical benefits and safety in patients with heart failure, and class Ic antiarrhythmic medications are generally avoided due to concerns for proarrhythmic/negative inotropic effects in patients with heart failure. Therefore, we propose that carvedilol can be selected as the preferred medication for the treatment of OT PVCs in patients with heart failure. In addition, it can be used safely when the patient’s heart function is not evaluated.

Meanwhile, a recent study suggested that flecainide also has an RyR2-inhibitory effect which can suppress SOICR [26]. Moreover, beta-blockers are known to be effective at reducing PVC symptoms by diminishing post-extrasystolic potentiation. From this point of view, the combination regimen of carvedilol and flecainide can be an effective treatment option for idiopathic OT PVCs.

### 4.6. Dosage of the Drugs

In an in vitro study, the concentrations of carvedilol required to suppress the SOICR (0.3–1 μM) were much higher than those needed for a beta-blockade (~1 nM) [27]. Therefore, a potent SOICR inhibition would require high doses of carvedilol, which could produce significant hypotension and bradycardia [28]. However, it has been reported that carvedilol has a high degree of lipophilicity and a large volume distribution. Thus, it accumulates at a higher concentration in the cardiac muscle than in the plasma [29,30,31]. Furthermore, the longer the exposure to carvedilol, the smaller the dose of carvedilol needed to inhibit the SOICR [7]. Pharmacologically, separating the beta-blocking and anti-SOICR activities of carvedilol could be one solution [7].

In our study, the carvedilol doses administered were as follows: 8 mg in 22 patients, 16 mg in 27, 32 mg in 2, and 64 mg in 1. Most patients used 8 mg or 16 mg, which are relatively low doses, and the degree of the reduction in the PVC burden did not significantly differ between these two doses: carvedilol 8 mg −6.1 ± 6.4% and carvedilol 16 mg −5.4 ± 11.5%, *p* = 0.790). In the flecainide group, a standard/high dose was used in 28% of the patients (15/53, 4 patients used 150 mg and 11 patients used 200 mg). The differences in drug dosages between the two drugs may have affected our experimental results. Of note, there was no difference in PVC burden reduction between the groups using 8 mg and 16 mg of carvedilol; the exact reason for this is unclear. Indeed, on the other hand, it is noteworthy that even at lower doses, carvedilol showed some antiarrhythmic effects. This suggests that lower doses of carvedilol may still be effective in treating OT PVCs and may have potential benefits in reducing the risk of adverse events associated with higher doses. Further studies are needed to determine the optimal dose of carvedilol for this indication.

### 4.7. Study Limitations

To date, many research results have been published on the mechanism of OT PVCs and pharmacological/non-pharmacological treatment methods. However, there are still unresolved questions: (1). What is the mechanism of OT PVCs that occurs independently of sympathetic activation, such as during rest or sleep? (2). Why does DAD occur only in the OT myocardium due to sympathetic activation? Moreover, is there a problem with the RyR2 in the OT myocardium, or can other receptors’ abnormalities also occur in the OT myocardium? (3). Does carvedilol only act on abnormal RyR2, or could it also act on normal RyR2? Interestingly, to further complicate the issue, as described in the study by Tang et al. [15], a 32.7% reduction in PVC burden was observed in the group that was only observed without treatment. This suggests that even frequent PVC patients have the possibility of spontaneous reduction in PVCs. It is believed that ongoing research on these questions is necessary, and one or more of these factors could explain the modest effect of carvedilol on OT PVCs in our study.

We used 24 h Holter monitoring to assess PVC frequency in our study, but recent evidence has demonstrated that substantial daily variation may occur [32]. Therefore, prolonged ECG monitoring may be required to quantify PVC frequency accurately. Currently, many ambulatory ECG monitoring modalities with capabilities of long-term ECG monitoring are already in use. Therefore, future PVC research should consider these aspects.

One of the significant limitations of our study is that we did not strictly control drug dosages and left them to the physicians’ discretion. This could have led to variations in the efficacy of the drugs, and the interpretation of the results should take this into account. Indeed, the reasons for determining the maximally tolerated dose should have been investigated in detail. Notably, a relatively low dose of carvedilol was used compared to flecainide. One possible explanation could be that physicians may have chosen to use lower doses to avoid potential side effects such as hypotension or bradycardia, which can occur with higher doses of carvedilol. This may have affected the research results, but it should be noted that while lower doses may have been used in some patients for the reasons mentioned above, the optimal dose of carvedilol for the treatment of OT PVCs has not yet been established and may vary depending on individual patient characteristics. Therefore, further studies are needed to determine the most effective and safe dosage of carvedilol for this indication.

Finally, although we performed randomization, this study was a pilot study with a small sample size, making it difficult to draw definitive conclusions.

## 5. Conclusions

In our study, both carvedilol and flecainide effectively suppressed idiopathic OT PVCs, but there was a difference in the degree of PVC suppression between the two drugs. We hope that our study results can contribute to further research in this field and encourage more comprehensive studies with larger sample sizes and stricter control over drug dosages.

## Figures and Tables

**Table 1:** Baseline characteristics.

Characteristic	Carvedilol (*n* = 51)	Flecainide (*n* = 52)	*p*-Value
Male	17 (33.3)	13 (25.0)	0.352
Age (y)	53.1 ± 12.6	55.5 ± 12.9	0.330
BMI (kg/m^2^)	24.3 ± 3.5	23.6 ± 3.0	0.282
SBP (mmHg)	120.82 ± 12.37	125.75 ± 17.01	0.096
DBP (mmHg)	73.06 ± 10.72	75.60 ± 13.79	0.300
Heart rate (/min)	73.20 ± 13.87	70.75 ± 10.08	0.308
History of hypertension	24 (47.1)	18 (34.6)	0.199
History of diabetes	4 (7.8)	3 (5.8)	0.715
Concomitant medications			
ACEI/ARB	18 (35.3)	12 (23.1)	0.172
Non-dihydropyridine CCB	11 (21.6)	8 (15.4)	0.419
Statins	14 (27.5)	14 (26.9)	0.952
Diuretics	2 (3.9)	1 (1.9)	0.618
LV EF (%)	61.0 ± 5.2	60.6 ± 3.8	0.675
LA dimension (cm)	3.73 ± 0.47	3.67 ± 0.48	0.367
Dosage of medication *			
	8 mg (21)	50 mg (1)	
	16 mg (27)	100 mg (36)	
	32 mg (2)	150 mg (4)	
	64 mg (1)	200 mg (11)	

Values are presented as the *n* (%) or mean ± SD. BMI: body mass index, SBP: systolic blood pressure, DBP: diastolic blood pressure, ACEI: angiotensin conversion enzyme inhibitor, ARB: angiotensin receptor blocker, CCB: calcium channel blocker, LV; left ventricle, EF; ejection fraction, LA; left atrium. * Sustained release formulation of carvedilol 8/16/32 mg, once-daily dose is equivalent to 3.125/6.25/12.5 mg twice daily.

**Table 2:** Blood pressure and electrocardiographic changes after three months of treatment.

Characteristic	Baseline	After 3 Months	*p*-Value
Carvedilol group (*n* = 51)			
Systolic blood pressure (mmHg)	121.76 ± 12.32	121.89 ± 14.29	0.7250
Diastolic blood pressure (mmHg)	73.06 ± 10.52	73.16 ± 10.57	0.8619
Heart rate (beats per minute)	77.16 ± 14.48	70.48 ± 12.83	0.0021
PR interval (ms)	160.03 ± 21.04	164.44 ± 23.83	0.004
QRS duration (ms)	89.71 ± 12.45	89.98 ± 13.14	0.6667
QTc interval (ms)	440.32 ± 38.25	434.46 ± 33.89	0.1760
Flecainide group (*n* = 52)			
Systolic blood pressure (mmHg)	125.08 ± 17.07	124.41 ± 13.52	0.5033
Diastolic blood pressure (mmHg)	74.48 ± 13.33	73.57 ± 11.09	0.7687
Heart rate (beats per minute)	75.41 ± 15.10	71.24 ± 9.78	0.2392
PR interval (ms)	160.18 ± 27.8	176.81 ± 28.92	<0.0001
QRS duration (ms)	92.49 ± 12.70	99.06 ± 13.03	<0.0001
QTc interval (ms)	439.13 ± 35.69	437.91 ± 29.36	0.6718

**Table 3:** Efficacy on PVC reduction according to each dosage of the drugs.

**Carvedilol**	8 mg SR * (*n* = 21)	**16 mg SR (*n* = 27)**	**32 mg SR (*n* = 2)**	**64 mg SR** **(*n* = 1)**
PVC burden reduction ≥ 10%	16	16	2	0
PVC burden reduction ≥ 50%	7	4	1	0
**Flecainide**	**50 mg (*n* = 1)**	**100 mg (*n* = 36)**	**150 mg (*n* = 4)**	**200 mg (*n* = 11)**
PVC burden reduction ≥ 10%	1	29	4	10
PVC burden reduction ≥ 50%	1	24	4	9

Presented number is the number of patients. PVC: premature ventricular complex. * Sustained release formulation of carvedilol 8/16/32 mg, once-daily dose is equivalent to 3.125/6.25/12.5 mg twice daily.

**Table 4:** Primary and secondary outcome measurements.

Characteristic	Carvedilol (*n* = 51)	Flecainide (*n* = 52)	*p*-Value
Medication compliance * (%)	96.3 ± 7.6	93.6 ± 9.4	0.095
Overall patients			
24 h PVC burden—baseline	20.3 ± 11.5	17.1 ± 9.9	0.191
24 h PVC burden—3 months after Tx	14.6 ± 10.8	6.6 ± 9.9	<0.0001
Mean difference of PVC burden	−5.6 ± 9.3	−10.6 ± 12.1	0.023
VAS—baseline	4.2 ± 2.1	4.1 ± 2.2	0.798
VAS—3 months after Tx	2.1 ± 2.2	2.0 ± 2.0	0.985

Values are presented as the *n* (%) or mean ± SD. * Adherence was assessed using the percentage of prescribed doses taken calculated as follows: number of doses taken/number of doses expected to be taken from the last prescription × 100 (%). PVC: premature ventricular complex, Tx: treatment, VAS: visual analogue scale.

## Data Availability

The datasets generated during and/or analyzed during the current study are available from the corresponding author upon reasonable request.
